# A case of obstructive jaundice caused by metastasis of breast cancer to the intra/extrahepatic bile duct

**DOI:** 10.1002/deo2.144

**Published:** 2022-06-20

**Authors:** Yuka Suzuki, Koki Hoshi, Keiichi Tominaga, Yasunori Inaba, Tomonori Yoshinaga, Shunsuke Kojimahara, Ryuichi Maki, Rena Nemoto, Yugo Tetsuka, Yosuke Kawata, Akira Yamamiya, Takeshi Sugaya, Yukihiro Iso, Atsuko Takada‐Owada, Kazuyuki Ishida, Kenichi Goda, Atsushi Irisawa

**Affiliations:** ^1^ Department of Gastroenterology Dokkyo Medical University School of Medicine Tochigi Japan; ^2^ Department of Hepato‐Biliary‐Pancreatic Surgery Dokkyo Medical University School of Medicine Tochigi Japan; ^3^ Department of Diagnostic Pathology Dokkyo Medical University School of Medicine Tochigi Japan

**Keywords:** breast cancer, endoscopic retrograde cholangiopancreatography, metastatic bile duct cancer, obstructive jaundice

## Abstract

A 38‐year‐old woman was admitted to our hospital for a detailed examination of jaundice. Three years before, she had undergone a right total mastectomy and axillary lymph node dissection of her right breast because of cancer. Histopathological evaluation revealed invasive ductal carcinoma. Postoperatively, because multiple bone metastases were found, she underwent chemoradiotherapy. Endoscopic retrograde cholangiopancreatography was performed, which revealed widespread multiple stenoses with a smooth surface from the intrahepatic to the extrahepatic bile duct. A transpapillary biliary biopsy was performed. Histopathological examination revealed adenocarcinoma extending into the subepithelium of the bile duct. The obtained cancer cells were similar to those of the earlier invasive breast cancer. This rare case demonstrates bile duct metastasis of breast cancer with specific endoscopic retrograde cholangiopancreatography findings.

## INTRODUCTION

Most distant metastases of breast cancer, an extremely common malignancy in women, occur in the lungs, bones, lymph nodes, liver, and brain. Although virtually every site of the human body can be targeted by the hematogenous spread of breast cancer, metastases are rare for intra‐abdominal organs other than the liver. We herein report a rare case demonstrating bile duct metastasis of breast cancer with specific endoscopic retrograde cholangiopancreatography (ERCP) findings.

## CASE REPORT

A 38‐year‐old woman was admitted to our hospital for a detailed examination of jaundice. She had undergone right total mastectomy and axillary lymph node dissection for breast cancer three years prior (clinical stage IIIc). Histopathological evaluation revealed invasive adenocarcinoma, negative margins, lymphatic involvement, and estrogen receptor positivity. Postoperatively, multiple bone metastases were found, for which she underwent chemoradiotherapy.

**FIGURE 1:**
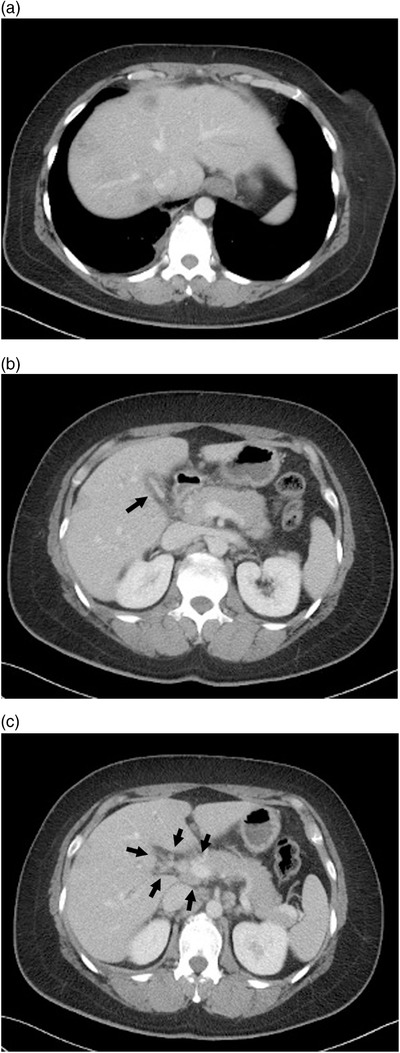
Contrast‐enhanced computed tomography. (a) Multiple intrahepatic low‐contrast density masses. (b) The gallbladder was shrunk and edematous with a thickened wall (arrow). (c) Enlarged hilar lymph nodes of the liver (arrow)

**FIGURE 2:**
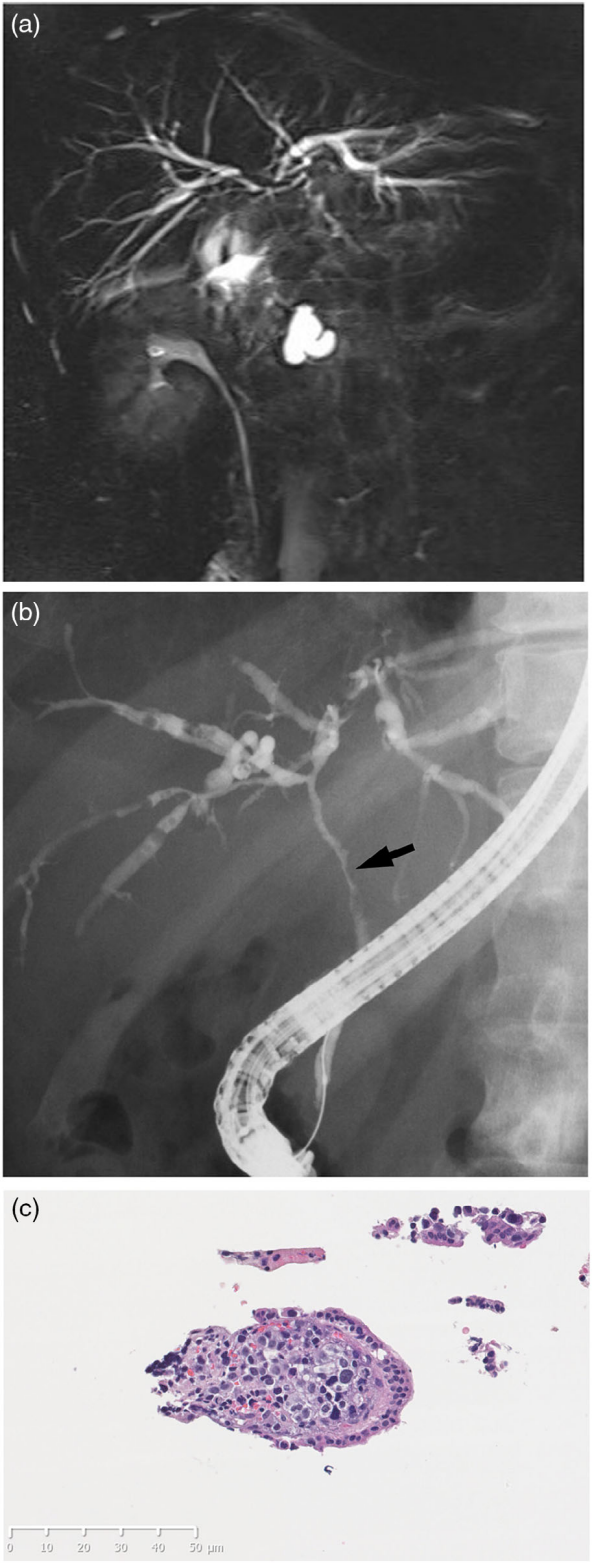
(a) Magnetic resonance cholangiopancreatography. There was extensive stenosis from the distal bile duct to the hilar bile duct and mild intrahepatic bile duct dilatation. (b) Endoscopic retrograde cholangiopancreatography showed extensive bile duct stenosis extending from the hilar to the extrahepatic side. The stenosis surface was smooth. The biopsy site is shown by an arrow. (c) Histopathological examination revealed adenocarcinoma extending into the subepithelium of the bile duct. Hematoxylin and eosin staining, original magnification, ×400

**FIGURE 3:**
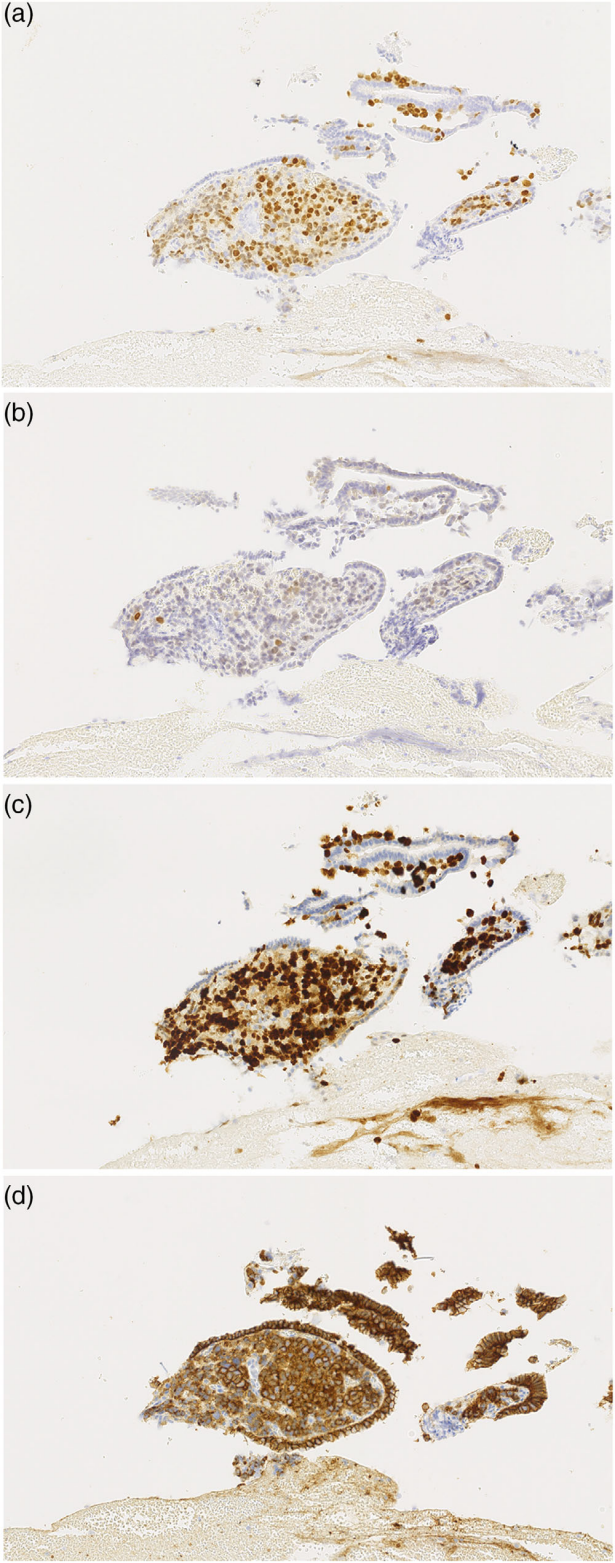
Histopathological findings from the hilar stenotic bile duct biopsy. The obtained cancer cells were similar to those of the earlier invasive breast cancer following immunohistochemical staining: (a) estrogen receptor‐positive, (b) progesterone receptor‐positive, (c) Ki‐67 positive, and (d) E‐cadherin positive

At the time of the visit, no enlarged superficial lymph node was found; no abnormal finding was obtained. Blood biochemical examination showed elevated hepatobiliary enzymes: AST 125 U/L, ALT 189 U/L, γ‐GTP 552 U/L, and T‐Bil 3.4 mg/dl. Tumor markers were also elevated: CEA 48.8 ng/ml, PIVKA2 104 mAU/ml, NCC‐ST‐439 670 U/ml, CA15‐3 51.0 U/ml, and CSLEX 1400 U/ml. Contrast‐enhanced computed tomography showed multiple intrahepatic low‐contrast density masses. The walls of intrahepatic and extrahepatic bile ducts showed contrast effects. Based on these findings, inflammation or cholangiocarcinoma was suspected (Figure 1a–c). Magnetic resonance cholangiopancreatography and ERCP showed widespread multiple stenoses with a smooth surface from the intrahepatic to the extrahepatic bile duct. These cholangiographical findings suggest the possibility of malignant stenosis (Figure 2a–b). However, these findings were regarded as different from the image of stenosis caused by an epithelial tumor. Consequently, the stenosis was inferred as attributable to subepithelial compression resulting from bile ductal metastasis of the earlier breast cancer, although cholangiocarcinoma could not be discounted as a possibility. We performed a transpapillary biopsy from the hilar stenotic bile duct using small forceps (1.8 mm, Radial Jaw 4P; Boston Scientific Corp., Boston, MA, USA), and a 7‐Fr plastic stent was placed. Histopathological examination revealed adenocarcinoma extending into the subepithelium of the bile duct (Figure 2c). The obtained cancer cells resembled those of the earlier invasive breast cancer, with the following immunohistochemical staining results: estrogen receptor‐positive, progesterone receptor‐positive, Ki‐67 positive, and E‐cadherin positive (Figure 3a–d). Based on these findings, we diagnosed bile ductal subepithelial metastasis of the earlier breast cancer. In this case, obstructive jaundice worsened because of extensive bile duct stenosis, even after stenting. The computed tomography images suggest enlarged hilar and periaortic lymph nodes. In addition, the presence of disseminated nodules is suspected based on elevated lipid concentrations in the regions of the hilum and around the gallbladder. It was regarded as difficult to continue chemotherapy. She received palliative care thereafter.

## DISCUSSION

Many cases of obstructive jaundice caused by liver metastasis from breast cancer have been reported. However, its major pathology is compression of the bile ducts by the tumor or tumor infiltration into the bile ductal wall. Direct metastatic involvement of the extrahepatic bile ducts in absence of hepatic lesions is exceptional.^1^, ^2^, ^3^ Similarly to our study, several reports have described direct metastasis to the bile ductal wall, although most of the patients had undergone surgical treatments such as cholangioduodenectomy or pancreatoduodenectomy for malignant bile duct stenosis of unknown origin.^4^, ^5^ Few case reports have described a definitive diagnosis reached before surgery. Budmir et al. reported a case of bile duct metastasis of breast cancer diagnosed based on cytology under ERCP. The patient was treated with endocrine therapy.^6^ However, this reported case demonstrated invasion into the bile duct from lymph node metastasis of breast cancer, which differs from direct metastasis to the bile ductal wall. In the presented case, results of cholangiography showed widespread multiple stenoses with a smooth surface from the intrahepatic to the extrahepatic bile duct. The findings differed from those found in common cholangiocarcinoma. Akiyama et al. reported extensive biliary stricture with smooth surfaces as a cholangiographic finding in metastatic biliary tumors.^7^ Moreover, Tang et al. reported that endoscopic ultrasound for metastatic bile duct tumor revealed a widening of the bile duct wall.^8^ Unfortunately, endoscopic ultrasound/intraductal ultrasound was not performed in this case, but endoscopic ultrasound/intraductal ultrasound often produces findings similar to those for gastrointestinal subepithelial tumors, which might provide a modality to facilitate diagnosis. Furthermore, secondary sclerosing cholangitis should be regarded as a possible differential diagnosis for such findings.^9^, ^10^ Secondary sclerosing cholangitis is regarded as resulting from radiation or drug‐induced adverse events, but in this case, secondary sclerosing cholangitis was ruled out because the irradiation was limited to bone metastases. In addition, some reports have described secondary sclerosing cholangitis caused by bevacizumab, paclitaxel, and pembrolizumab, but those drugs were not used by this patient.

The histopathological examination showed that adenocarcinoma mainly infiltrated into the subepithelium of the bile duct. Therefore, we inferred that ERCP findings indicating smooth bile duct stenosis would reflect the pathology of the metastatic tumor growth. This patient showed metastasis to the peripheral side of the liver. We considered the possibility of hematogenous metastasis to the bile duct via the peribiliary capillary plexus based on the following findings: (1) the liver metastasis was peripheral to the liver, whereas the main site of bile duct stricture was from the hilar region to the upper bile duct; (2) magnetic resonance cholangiopancreatography and ERCP findings showed no evidence of direct bile duct obstruction caused by liver metastasis; and (3) histopathological findings showed no evidence of bile duct obstruction. Moreover, liver metastasis was regarded as secondary to bile duct metastasis. However, simultaneous metastasis to the bile duct and liver cannot be ruled out completely. It should be kept in consideration.

Direct metastasis of breast cancer to the bile duct is extremely rare. Metastatic bile ductal tumors, like those examined in this study, might present with smooth stenosis, suggesting the presence of normal epithelium of the bile duct. It is noteworthy that aspiration cytology of the bile juice and brush cytology might give false‐negative results in cases with similar stenosis. When performing a biopsy under ERCP in similar cases, it is necessary to perform a biopsy so as to obtain not only the epithelium but also the stroma.

## CONFLICT OF INTEREST

The authors declare that they have no financial relationship associated with this case report and the study it describes.

## FUNDING INFORMATION

None.

## ETHICS STATEMENT

This case report was conducted in accordance with the ethical standards established in the 1964 Declaration of Helsinki and its later amendments.
